# Understanding weight status and dietary intakes among Australian school children by remoteness: a cross-sectional study

**DOI:** 10.1017/S1368980023000198

**Published:** 2023-06

**Authors:** Jane Jacobs, Claudia Strugnell, Denise Becker, Jill Whelan, Josh Hayward, Melanie Nichols, Andrew Brown, Victoria Brown, Steven Allender, Colin Bell, Andrew Sanigorski, Liliana Orellana, Laura Alston

**Affiliations:** 1 Global Obesity Centre (GLOBE), Institute for Health Transformation, Deakin University, Waterfront Campus, 1 Gheringhap St, Geelong, VIC 3220, Australia; 2 Deakin University, Biostatistics Unit, Faculty of Health, Geelong, Australia; 3 Deakin University, Deakin Health Economics, Institute for Health Transformation, Faculty of Health, Geelong, Australia; 4 Deakin Rural Health, School of Medicine, Faculty of Health, Deakin University, Geelong, Australia

**Keywords:** Dietary intake, Rural health, Childhood obesity, Health inequalities

## Abstract

**Objective:**

To determine whether primary school children’s weight status and dietary behaviours vary by remoteness as defined by the Australian Modified Monash Model (MMM).

**Design:**

A cross-sectional study design was used to conduct secondary analysis of baseline data from primary school students participating in a community-based childhood obesity trial. Logistic mixed models estimated associations between remoteness, measured weight status and self-reported dietary intake.

**Setting:**

Twelve regional and rural Local Government Areas in North-East Victoria, Australia.

**Participants:**

Data were collected from 2456 grade 4 (approximately 9–10 years) and grade 6 (approximately 11–12 years) students.

**Results:**

The final sample included students living in regional centres (17·4 %), large rural towns (25·6 %), medium rural towns (15·1 %) and small rural towns (41·9 %). Weight status did not vary by remoteness. Compared to children in regional centres, those in small rural towns were more likely to meet fruit consumption guidelines (OR: 1·75, 95 % CI (1·24, 2·47)) and had higher odds of consuming fewer takeaway meals (OR: 1·37, 95 % CI (1·08, 1·74)) and unhealthy snacks (OR = 1·58, 95 % CI (1·15, 2·16)).

**Conclusions:**

Living further from regional centres was associated with some healthier self-reported dietary behaviours. This study improves understanding of how dietary behaviours may differ across remoteness levels and highlights that public health initiatives may need to take into account heterogeneity across communities.

Australian data shows children living in regional and rural areas are more likely to be affected by overweight or obesity compared with those living in major cities^(1)^, with the difference widening throughout adulthood^(2)^. This trend is also apparent in other high-income countries^(3–5)^. Children who experience overweight or obesity are more likely to have asthma, musculoskeletal issues, CVD and poor dental health^(6)^, a pattern shown to persist from childhood to adulthood^(7)^. A key driver of global obesity is poor dietary intake^(8)^. A majority of Australian children do not consume a diet consistent with the Australian Dietary Guidelines^(9)^. The most recent national survey (2017–2018) reported 73 % of Australian children and adolescents aged 2–17 meet fruit intake guidelines, while only 6·3 % meet vegetable intake guidelines^(10)^. Children and adolescents’ intake of discretionary foods has increased substantially in recent decades, and these foods now contribute approximately 40 % of young Australian’s daily energy intake^(11)^, while 47 % of boys and 35 % of girls consume at least one sugar-sweetened beverage (SSB) per week^(10)^.

Most dietary intake studies in Australia, and internationally, dichotomise children into binary classifications with those living in major cities areas and those living outside of major cities (generally termed regional, rural or remote)^(3,12–14)^.This has been a major limiting factor in generating evidence to improve health in regional, rural and remote areas. It is apparent that health inequities are persistent between major city and regional/rural populations, and modifiable risk factors, such as diet, are known to contribute to these disparities^(15,16)^. Areas outside of major cities can vary greatly by population size and density, availability of and access to, health-promoting resources, healthy food, population demographics, socio-economic position (SEP) and social norms, all of which can impact dietary patterns^(17–19)^. Categorising all populations outside of major cities together may miss detecting important differences across and between these regional and rural populations^(12,13)^. In addition, a lack of disaggregated rural data has also led to a lack of knowledge to inform dietary interventions in rural and regional areas, potentially exacerbating health disparities^(20,21)^.

Encouraging healthy dietary patterns in childhood can reduce the risk of obesity and risk of non-communicable diseases in adulthood and presents as a key opportunity to address and prevent long-term health disparities between metropolitan and rural populations^(22)^. Current Australian dietary guidelines recommend children consume at least two serves of fruit and five vegetables each day (5·5 for boys over 12)^(9)^, which is similar to guidelines published in other high-income countries^(23,24)^. Discretionary food includes products containing high levels of saturated fat, added salt and added sugar, such as processed meats, commercial pizza, fried foods, potato chips, crisps and other savoury snacks and SSB^(9)^. Dietary guidelines recommend limiting the consumption of these foods, particularly in children^(9,23,24)^.

Investigations of dietary intake are needed that consider more nuanced variation across regional and rural locations. The measurement of remoteness varies considerably within and between countries^(25)^, which can result in difficulty in generating relevant evidence to inform policy for these populations. Since the late 1990s, remoteness in Australia has been determined by the Accessibility/Remoteness Index of Australia (ARIA), which classifies areas into five categories from major city through to very remote^(26)^. In 2015, the Modified Monash Model (MMM) was developed, primarily to address the issue of health workforce distribution^(27)^. Taking into consideration population size and geographic remoteness, the MMM provides an even more fine-grained categorisation of areas, with seven classifications of remoteness^(27)^. The UK^(28)^ and Finland^(29)^ use similar categorical gradient systems to define urban and rural locations, although they both have multiple urban classifications while Australia only has one ‘major city’ category. The MMM has been used increasingly in health-related research to address the paucity of research considering the heterogeneity of regional and rural Australia^(30)^. To date, there have been no studies, to our knowledge, that investigate children’s dietary intake using the MMM.

To add to the knowledge of how primary school children’s diet and weight status may vary with degree of remoteness, this study aims to determine if: (1) for the first time, to our knowledge, remoteness, as measured by the MMM, is associated with weight status and meeting fruit and vegetable intake guidelines; and (2) the consumption of discretionary foods and SSB varies by remoteness.

## Methods

This is a cross-sectional analysis of the baseline (2019) data from children participating in the obesity prevention cluster-randomised trial – the Reflexive Evidence and Systems interventions to Prevent Obesity and Non-communicable Disease (RESPOND) study. The trial was undertaken across twelve local government areas (two pilot and ten intervention) in North-East Victoria, Australia. The full details of the methods have been described elsewhere^(31)^.

### School and student recruitment

All primary schools within the twelve participating local government areas were invited to participate in the study by contacting the school principal or representative. Within consenting schools grade 2 (aged approximately 7–8 years) grade 4 (aged approximately 9–10 years) and grade 6 (aged approximately 11–12 years) children were invited to participate using a passive (opt-out) approach. Students were enrolled in the study unless an opt-out form signed by a parent or guardian was returned, or the student verbally declined to participate at the time of measurement and surveys being completed. Students could also choose to participate in all, or only some aspects of the study (e.g. only the survey).

### Anthropometry

The height and weight of grades 2, 4 and 6 students were measured according to a standardised protocol by trained staff^(31)^. Height was measured to the nearest 0·1cm, and weight was measured to the nearest 0·05 kg. Measurements were taken twice, and if there was a difference of greater than 0·1 kg or 0·5 cm, a third measure was taken. The mean of all measurements was used. Measurements were used to calculate BMI *Z*-scores (BMI-*Z*), which were categorised as overweight (+1 < BMI < 2) or obese (BMI-*Z* ≥ +2) based on the WHO growth reference^(32)^.

### Dietary intake

Dietary intake was assessed using sixteen self-report questions collated from three nutrition questionnaires. Only the grades 4 and 6 students completed these surveys. Fruit and vegetable serves were assessed with modified questions from the Child Nutrition Questionnaire^(33)^. Discretionary food, including unhealthy snacks (e.g. chips, lollies, chocolate, cakes, biscuits and pastries), SSB (e.g soft drink, fruit juice and cordial) and takeaway consumption, was examined using fourteen items from the Food, Health, and Choices Questionnaire^(34)^. Water consumption was assessed using one question from the Simple Dietary Questionnaire (NPJ Parletta, K O’Dea and C Itsiopoulos, unpublished results).

Questionnaire responses were used to create indicator variables on whether or not the student met the Australian Dietary Guidelines for daily serves of fruit and vegetables (≥2 serves of fruit, ≥5 serves of vegetables or ≥5·5 serves of vegetable for boys 12–18 years) and water intake (≥5 glasses/d)^(9)^. There are no current quantitative guidelines for SSB, takeaway or unhealthy snack consumption; therefore, cut-points were decided on by the researchers of <1/d/≥1/d for SSB; ≤once a fortnight/ >once a fortnight for takeaway and <1/d/ ≥1/d for unhealthy snack consumption.

### Remoteness

The primary measure used to classify geographical remoteness was the MMM that takes into account geographical remoteness and town size^(27)^. The current classification was released in 2019, based on the Australian Bureau of Statistics (ABS) 2016 Census data and assigns areas into seven ordinal categories from Modified Monash (MM) 1 (major city) through to MM7 (very remote)^(27)^. Modified Monash level was allocated according to students’ self-reported residential town and postcode. Town name was initially matched to ‘suburb/locality’ in the MMM database. Where town was missing, postcode was used if it was located within a single MM level. Where both town and postcode were missing, or postcode was located in multiple MM levels, the student was excluded from analysis. Student locations were spread across MM1 (metropolitan areas) to MM5 (small rural towns), with no students in MM6 (remote communities) or MM7 (very remote communities). Children in the MM1 (major city) category was excluded from analysis, as this group was too small to be an independent category (4·4 % of sample) but was considered too different from the next category (MM2 regional centres) for these groups to be combined.

### Socio-economic position

SEP was based on students’ home postcodes and measured according to the Index of Relative Socio-economic Advantage and Disadvantage (IRSAD)^(35)^. Based on ABS Census data for 2016, an IRSAD score is determined for each geographic area, taking into consideration individual measures of both advantage and disadvantage (e.g. education, employment, income, home ownership) for the resident population. For analysis, IRSAD scores were classified into Victoria-wide tertiles. While IRSAD is not theoretically connected to remoteness (there are no inputs associated with remoteness in the IRSAD model), a major city to very remote gradient is apparent, reflecting lower SEP as populations are further from major cities^(17)^.

### Statistical analysis

Only data from grades 4 and 6 students were included in the analysis as grade 2 students did not complete the dietary questionnaire. Descriptive analysis shows the demographics of the sample and distribution of weight status and dietary behaviours according to MMM categories. Mixed effect logistic regressions were fitted to estimate the associations between MMM category and weight status, meeting guidelines (fruit, vegetable and water intake), or having high/low consumption (SSB, takeaway, snacks). To account for confounding, adjustment was made for sex and grade. To further investigate the impact of SEP on the outcomes, a second model additionally adjusted for IRSAD. All models included school as a random effect to account for within-school clustering. A *P*-value of <0·05 was considered significant for all analyses. All analyses were conducted in Stata version 16.1.

Approximately 40 % of students resided in areas classified as MM5, therefore a supplementary analysis was undertaken to further explore the impact of remoteness on this group. Students in MM5 areas were divided into those classified as inner regional and those classified as outer regional areas according to the ARIA+ classification system^(36)^, creating a five level variable; MM2, MM3, MM4, MM5_RA2 (MM5/inner regional), MM5_RA3 (MM5/outer regional). The same regression models outlined above were run with this five-level variable.

## Results

Ninety-one out of 163 schools agreed to participate in baseline monitoring, representing a school-level participation rate of 55·8 %. Within these schools, 3889/4736 (82·1 %) of Grades 2, 4 and 6 students participated in data collection in 2019, with anthropometric and dietary behaviour data being collected from a total of 2601 grade 4 and 6 students. Twenty-two students were excluded due to missing postcode or binary gender data, and a further 114 were excluded as they resided in a MM1 location. The final sample included 2456 students (94·4 % of the original sample).

The largest proportion of students lived in small rural towns (41·9 %) and were in the lowest SEP category (64·9 %) (Table 1). Of the included sample, 36·2 % had overweight or obesity.

**Table 1:** Description of a sample of Australian school children (Grades 4 and 6) from rural and regional areas of north-east victoria (*n* 2465)

	Total		
	*N*	*n*	%
Demographics			
Female	2465	1212	49·2
Grade 6	2465	1168	47·4
Tertile of SEP*			
Lowest	2465	1600	64·9
Middle	2465	587	23·8
Highest	2465	278	11·3
Government school	2465	2156	87·5
Aboriginal or Torres Strait Islander	1994	202	10·1
Speak LOTE at home	2444	183	7·5
MMM remoteness (home town based)			
Regional centres (pop >50 000)	2465	429	17·4
Large rural towns (pop 15 000–50 000)	2465	630	25·6
Medium rural towns (pop 5000–15 000)	2465	372	15·1
Small rural towns (pop 1000–5000)	2465	1034	41·9
ARIA + remoteness areas (home town based)			
Major cities	2460	3	0·1
Inner regional	2460	2132	86·7
Outer regional	2460	325	13·2
Weight			
Overweight or obese (WHO classification)	2333	845	36·2
Meeting food/drink guidelines			
Vegetables (≥5/ 5·5 serves/d†)	2454	404	16·5
Fruit (≥2 serves/d)	2454	1793	73·1
Water (≥5 glasses/d)	2454	1363	55·5
Take away (≤once a fortnight)	2454	1480	60·3
Unhealthy snacks (< once/d)	2453	916	37·3
Sweetened drinks (< once/d)	2454	1397	56·9

*N* = total sample; *n* = sample meeting that criteria; SEP, socio-economic position; IRSAD, Index of Relative Socio-Economic Advantage and Disadvantage; LOTE, language other than English; MMM, Modified Monash Model; pop, population (approximate populations according to MMM classification)^(33)^.

* Student IRSAD VIC tertile – home postcode based. Lowest/ middle/highest Victoria wide tertiles of IRSAD (note NSW. postcodes assigned according to Vic tertile cut-offs).

† ≥ 5 serves/d for boys <12 and all girls, and ≥5.5 for boys ≥12 years old.

MMM classification was not associated with the prevalence of overweight or obesity (Table 2 and see online Supplemental Table 1). There were also no significant findings for meeting vegetable or water guidelines.

**Table 2:** Associations of MMM rurality with weight status and meeting food/drink consumption guidelines (*n* 2465)

Outcome	Rurality	Model 1				Model 2			
		OR	95 % CI	*P* *	*P* †	OR	95 % CI	*P* *	*P* †
Overweight or obese (WHO^(32)^)	MM2	Ref				Ref			
	MM3	1·27	0·87, 1·86	0·217		1·08	0·75, 1·53	0·689	
	MM4	1·02	0·68, 1·54	0·912		0·85	0·58, 1·25	0·400	
	MM5	0·94	0·68, 1·32	0·732	0·206	0·96	0·71, 1·31	0·809	0·568
Vegetables (≥ 5 or 5·5 serves/d*)	MM2	Ref				Ref			
	MM3	0·91	0·61, 1·37	0·658		0·89	0·59, 1·35	0·589	
	MM4	0·82	0·51, 1·30	0·394		0·79	0·49, 1·27	0·327	
	MM5	0·87	0·60, 1·26	0·417	0·844	0·87	0·60, 1·27	0·470	0·797
Fruit (≥ 2 serves/d)	MM2	Ref				Ref			
	MM3	1·42	0·97, 2·08	0·07		1·46	0·99, 2·15	0·055	
	MM4	0·97	0·64, 1·45	0·864		1·01	0·67, 1·54	0·954	
	MM5	1·74	1·24, 2·44	**0·001**	**0·001**	1·75	1·24, 2·47	**0·001**	**0·001**
Water (≥ 5 glasses/d)	MM2	Ref				Ref			
	MM3	1·18	0·87, 1·61	0·290		1·21	0·88, 1·65	0·239	
	MM4	1·18	0·84, 1·67	0·342		1·24	0·87, 1·76	0·236	
	MM5	1·15	0·87, 1·52	0·354	0·721	1·17	0·88, 1·55	0·276	0·608
Take away (≤1/fortnight)	MM2	Ref				Ref			
	MM3	0·92	0·71, 1·18	0·495		0·96	0·74, 1·24	0·746	
	MM4	1·08	0·81, 1·43	0·610		1·15	0·86, 1·54	0·338	
	MM5	1·43	1·12, 1·81	**0·003**	**<0·001**	1·37	1·08, 1·74	**0·010**	**0·008**
Unhealthy snacks (< 1/d)	MM2	Ref				Ref			
	MM3	1·06	0·74, 1·51	0·763		1·13	0·79, 1·62	0·515	
	MM4	1·07	0·72, 1·60	0·741		1·19	0·79, 1·78	0·411	
	MM5	1·56	1·13, 2·14	**0·006**	**0·002**	1·58	1·15, 2·16	**0·005**	**0·011**
Sweetened drinks (< 1/d)	MM2	Ref				Ref			
	MM3	0·72	0·48, 1·09	0·116		0·85	0·58, 1·24	0·392	
	MM4	0·75	0·48, 1·16	0·188		0·91	0·60, 1·38	0·657	
	MM5	1·12	0·78, 1·60	0·547	**0·008**	1·12	0·80, 1·56	0·517	0·278

MM, modified Monash; ref, reference category; MM2, Regional centres; MM3, Large rural towns; MM4, Medium rural towns; MM5, Small rural towns.

* Overall *P*-value.

† Global *P*-value.

From logistic mixed models, school as random effect. Model 1 adjusted for sex and grade; Model 2 adjusted for sex, grade and IRSAD tertile.

Bold type indicates results that were significant at *P* < 0·05.

**Fig. 1:**
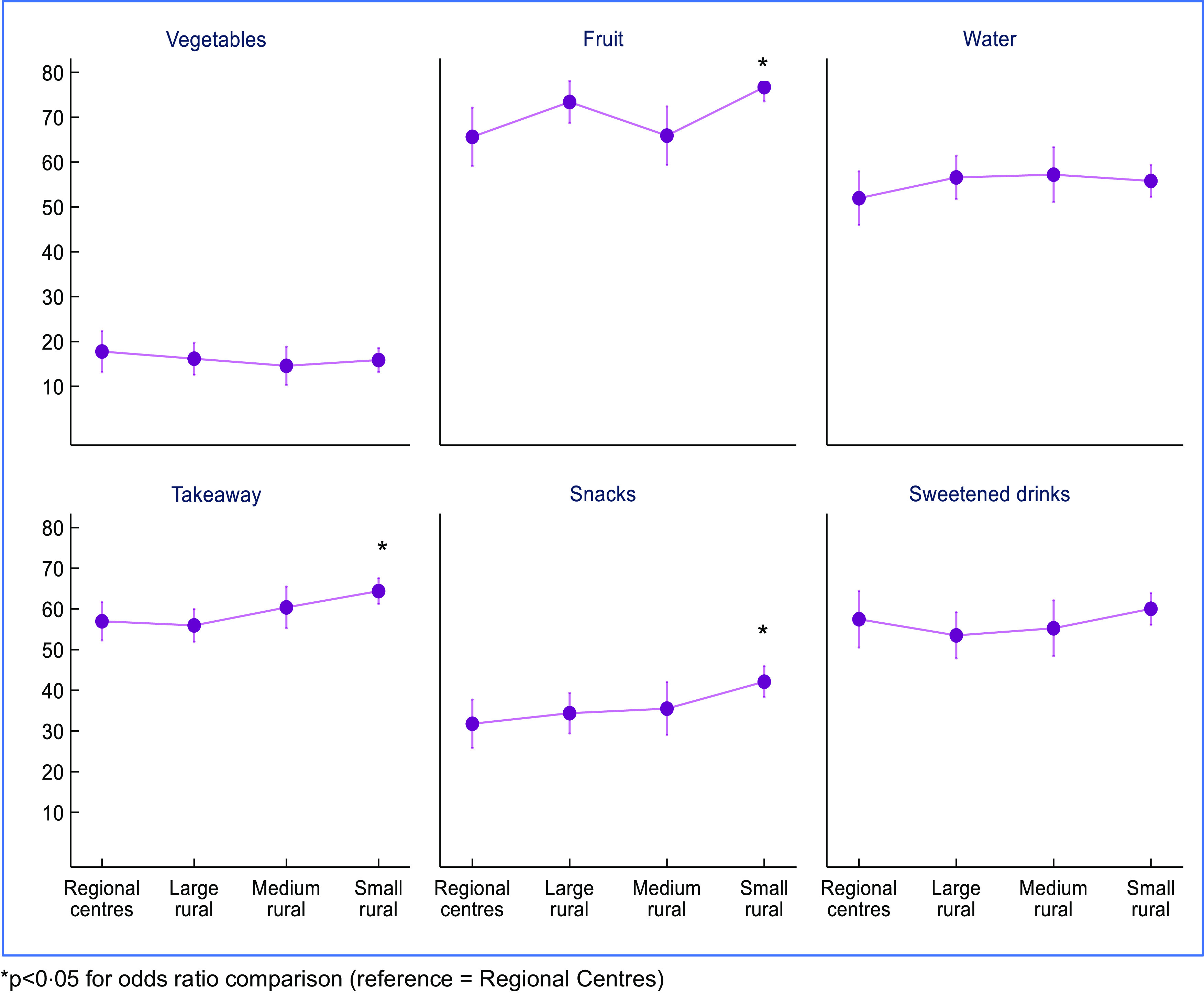
Outcomes from separate logistic mixed models, school as random effect, adjusted for sex, grade and IRSAD tertile. IRSAD, Index of Relative Socio-economic Advantage and Disadvantage

Table 2 shows that students in small rural towns (MM5) had 1·75 times the odds (OR 1·75; 95 % CI (1·24, 2·47)) of meeting fruit intake guidelines compared to those in regional centres (MM2), after adjustment for grade, sex and IRSAD. Students in small rural towns also had higher odds of having lower takeaway consumption (OR: 1·37, 95 % CI (1·08, 1·74)) and snack consumption (OR = 1·58, 95 % CI (1·15, 2·16)) compared to those living in regional centres in fully adjusted models (Fig. 1).

The breakdown of small rural towns into inner (MM5_RA2) and outer regional (MM5_RA3) categories showed similar pattens with a number of healthier behaviours being more prevalent among children living in small rural towns/outer regional areas (see online Supplemental Table 2 and Supplemental Fig. 1). The highest odds of meeting fruit consumption guidelines (OR 2·95, 95 % CI (1·85, 4·70)), low takeaway consumption (OR 1·89, 95 % CI (1·35, 2·65)) and low snack consumption (OR 2·25, 95 % CI (1·53, 3·32)) compared to students living in regional centres were recorded for small rural towns in outer regional areas.

## Discussion

This study is the first to analyse dietary behaviours of Australian children across fine-grained remoteness categories, taking into consideration the large heterogeneity of rural and regional areas using the MMM. The analysis found that students living in small rural towns were more likely to meet fruit consumption guidelines and have lower consumption of takeaway food and unhealthy snacks when compared to students in regional centres. In a supplementary analysis, these associations were found to be strongest for students living in small rural towns in outer regional areas. Weight status, meeting vegetable and water intake guidelines and SSB consumption did not vary with remoteness in fully adjusted models.

Recent reviews have highlighted significant gaps in rural dietary and food environment data in Australia and globally^(21,37,38)^ with a noticeable lack of dietary intervention research in rural communities in Australia^(20,39)^. Further, few interventions focussed on the food environment specific to rural areas have been conducted globally^(19)^, despite the evidence that rural food environments are less healthy^(40,41)^. In addition to a lack of research in these settings, rural communities tend to experience multiple factors that compromise food security, such as poor access to food (both due to price and physical access) and the relatively lower availability and higher cost of healthy food^(41)^. Such vulnerability is exacerbated in situations of crisis, such as that observed during the COVID-19 pandemic^(42)^.

However, our results show some healthier dietary behaviours as remoteness increased; and this may be linked to unknown factors within food environments or drivers of intakes in these areas. Our dietary results are broadly in line with the 2015 N.S.W Schools Physical Activity and Nutrition Survey (SPANS) report which also found rural children to be more likely to meet fruit guidelines than their major city counterparts^(12)^. They are also consistent with a UK study that found 10-year-old rural students were more likely to consume healthier diets compared to their major city counterparts^(43)^. In contrast, the most recent Australian National Health Survey (2017–2018) found no difference in meeting fruit guidelines between children living in major cities and outside of major cities^(1)^. These studies only presented binary results and did not further categorise children outside of major cities. Our study adds to the evidence by demonstrating intake patterns across varying levels of remoteness.

We did not find differences in weight across the included regional and rural categories in this Victorian sample, and we were not able to compare them against a major city cohort. The Australian Health Survey found higher rates of overweight and obesity reported in children living in regional and rural areas of Australia compared to their major city counterparts^(1)^. A bigger sample across all states of Australia may be why our results are different to the Australian Health Survey, alongside the Australian Health Survey not using the MMM in reporting differences by remoteness. A further Australian study, including over a million measures from children aged 3·5 and under, a different age range to what was investigated here, found obesity levels increased significantly from major city to outer regional areas^(44)^. While we found some healthier dietary behaviours the further children lived from major cities, which may appear counterintuitive given the weight distributions, we know that weight status is complex and impacted by many factors, beyond diet alone, and we have only explored a limited number of dietary factors in this study, using self-report measures.

The reduced takeaway consumption that was apparent in our results as remoteness increased may be due to the differing food environments. In particular, reduced availability of takeaway outlets the further a child lives from major cities and large regional centres may result in reduced opportunity for consumption. However, there remains conflicting evidence regarding the proximity of food outlets and dietary intake^(45)^. A census of food outlet types undertaken across medium (MM4) and small (MM5) rural Victorian towns showed twice as many local takeaways in the MM4 compared to MM5 category (10 *v*. 5), and five takeaway franchises in MM4 areas compared to none in MM5 areas^(40)^. An additional study of rural food environments in regional and rural Victoria found there to be poor access to healthy food in these areas^(41)^. In another regional area of Victoria, although not explored by remoteness, no associations were found between children’s dietary intake and the school food environment^(22)^.

Our study did not find any associations between remoteness and meeting vegetable consumption guidelines, although the overall proportion meeting vegetable consumption guidelines was higher in our sample than in previous surveys^(10)^. Across Australian populations, children’s vegetable consumption is low (6·3 % meeting guidelines), and this does not appear to vary significantly between major city and regional/rural locations^(10)^. International studies have found higher vegetable intakes in more rural and agricultural regions, with links to production and access to farm produce as factors in increasing intakes in rural compared to urban areas^(46)^. Further exploration is needed in areas of higher remoteness (such as small rural towns) to understand the facilitators of higher consumption in these communities.

With broad classification systems, particularly those that dichotomise the geographical location into major cities and regional/rural areas, the relatively greater populations in larger regional centres may primarily drive results. This could result in obscuring the outcomes of the smaller rural locations, and this has been a limiting factor in evidence on regional, rural and remote populations to date. Our study highlights the heterogeneity in rural and regional settings that can be captured when applying the MMM classification system which breaks down regional and rural areas to a greater extent that previously used measures such as a dichotomised approach or other standardised measures of remoteness. Applying consistent and meaningful classification of rurality in health research is an ongoing issue both in Australia and internationally^(13,25)^, and a lack of consistent definitions may hamper attempts at targeting public health interventions and policies appropriately. Moving beyond the dichotomisation of major city and outside of major city in health research has been called for in the literature^(47)^, however has rarely been implemented. Application of the MMM system has great potential to identify the needs of different regional and rural populations and enable tailoring of public health messaging and recommendations accordingly, in-part addressing heterogeneity across regional and rural communities. Our study highlights the significant variations in behaviours across regional and rural populations, which can potentially have differential impacts on children’s health outcomes and future patterns in non-communicable disease burden in these areas.

A major strength of this study is that we had a large sample of students outside of major cities, providing the opportunity to break regional and rural students into a further five categories, which has not been done in an Australian sample previously. A further strength of this study is the use of measured height and weight by trained researchers, providing accurate overweight and obesity estimates. The use of the standardised MMM to explore patterns by remoteness increases the generalisability of these findings to other similarly categorised regional and rural areas in Australia and provides new knowledge on patterns in children’s dietary intakes across remoteness levels.

A number of limitations also need to be acknowledged. While we achieved a large sample size of children in rural and regional areas, we did not have children from major cities in the analysis to show contrasts between these students and those living outside of major cities. This also limits our ability to compare these results to past research. Additional limitations relate to the use of self-report questionnaires to determine dietary intake, with issues regarding social desirability bias and interpretation of self-report questionnaires for children^(48)^. While these questionnaires used have acceptable reliability and validity in this age group (NPJ Parletta, K O’Dea and C Itsiopoulos, unpublished results)^(33,34)^, only some parts of the overall questionnaires were used, which may impact the interpretability of the results. Self-report outcomes have the potential for over and under-reporting, which may have contributed to higher reporting of meeting vegetable guidelines in our study compared to national surveys^(10)^. Further, being a cross-sectional study, we are not able to infer causation between residential location and weight status or dietary behaviours.

Data were examined as categorical rather than continuous variables, which may limit our ability to identify more nuanced results across remoteness levels. This approach was taken due to the nature of the questions that were asked to the children and how they were likely to be interpreted, along with many answer options being multiple choice only. Defining whether children meet guidelines (yes/no) also makes this study more comparable to previous research^(22)^ and provides relevant detail for comparison with public health recommendations^(9)^.

## Conclusion

This study is the first to examine the variation in dietary intake in children across the regional and rural spectrum in the Australian context. Weight outcomes were not associated with remoteness; however, generally healthier dietary behaviours were identified the further children lived from major cities. Further exploration is needed to understand why children in areas classed as small rural towns are more likely to report healthier diets than their less remote counterparts, in order to inform improvements across all levels of remoteness. The use of multiple categories across the remoteness spectrum allowed for a nuanced approach to analysis and highlighted the need to increase awareness of the heterogeneity of regional and rural areas and apply public health initiatives accordingly.

## Supplementary material

For supplementary material accompanying this paper visit https://doi.org/10.1017/S1368980023000198. click here to view supplementary material
