# Supplementary material for: Understanding weight status and dietary intakes among Australian school children by remoteness: a cross-sectional study
Source: Public Health Nutr. 2023 Jan 30;26(6):1185–93. doi: 10.1017/S1368980023000198 (PMC10346081; doi:10.1017/S1368980023000198)
Supplement: Supplementary file 1 [file S1368980023000198sup001.docx]

***Supplementary Table 1:*** Proportion of students meeting food/drink consumption guidelines (N = 2,465)

| **Outcome** | **Rurality** | **N** | **n** | **adj %** | **(95 % CI)** |  |
| --- | --- | --- | --- | --- | --- | --- |
| Overweight or obese (WHO) | MM2 | 400 | 151 | 37.4 | (31.4 , 43.5) |  |
|  | MM3 | 579 | 231 | 39.1 | (33.8 , 44.4) |  |
|  | MM4 | 354 | 126 | 33.7 | (27.7 , 39.7) |  |
|  | MM5 | 1000 | 337 | 36.6 | (32.9 , 40.3) |  |
| Vegetables (≥ 5 or 5.5 serves/day*) | MM2 | 427 | 82 | 17.8 | (13.1 , 22.4) |  |
|  | MM3 | 629 | 104 | 16.2 | (12.6 , 19.7) |  |
|  | MM4 | 371 | 55 | 14.6 | (10.3 , 18.9) |  |
|  | MM5 | 1027 | 163 | 15.9 | (13.2 , 18.5) |  |
| Fruit (≥ 2 serves per day) | MM2 | 427 | 291 | 65.6 | (59.1 , 72.2) |  |
|  | MM3 | 630 | 465 | 73.4 | (68.7 , 78.1) |  |
|  | MM4 | 370 | 245 | 65.9 | (59.4 , 72.4) |  |
|  | MM5 | 1027 | 792 | 76.7 | (73.6 , 79.9) |  |
| Water (≥ 5 glasses per day) | MM2 | 427 | 226 | 52.0 | (46.0 , 57.9) |  |
|  | MM3 | 630 | 360 | 56.6 | (51.7 , 61.4) |  |
|  | MM4 | 371 | 209 | 57.2 | (51.1 , 63.3) |  |
|  | MM5 | 1026 | 568 | 55.8 | (52.2 , 59.4) |  |
| Take away (≤1 per fortnight) | MM2 | 427 | 243 | 57.0 | (52.3 , 61.7) |  |
|  | MM3 | 629 | 347 | 56.0 | (52.0 , 60.0) |  |
|  | MM4 | 371 | 219 | 60.4 | (55.3 , 65.5) |  |
|  | MM5 | 1027 | 671 | 64.4 | (61.2 , 67.5) |  |
| Unhealthy snacks (< 1 per day) | MM2 | 426 | 139 | 31.8 | (25.9 , 37.7) |  |
|  | MM3 | 629 | 217 | 34.4 | (29.4 , 39.4) |  |
|  | MM4 | 371 | 120 | 35.5 | (29.0 , 42.0) |  |
|  | MM5 | 1027 | 440 | 42.1 | (38.3 , 45.9) |  |
| Sweetened drinks (< 1 per day) | MM2 | 427 | 235 | 57.5 | (50.5 , 64.4) |  |
|  | MM3 | 629 | 328 | 53.5 | (47.9 , 59.2) |  |
|  | MM4 | 371 | 193 | 55.2 | (48.4 , 62.1) |  |
|  | MM5 | 1027 | 641 | 60.0 | (56.1 , 63.9) |  |
| Adjusted percentages are from logistic mixed models, school as random effect, adjusted for gender, grade and IRSAD tertiles  MM2: Regional centres; MM3: Large rural towns; MM4 Medium rural towns; MM5: Small rural towns; WHO: World Health Organization; MM: Modified Monash | | | | | | |

| ***Supplementary Table 2:*** Associations of 5 level MM_RA rurality with meeting food/drink consumption guidelines (N = 2,462) | | | | | | | | | | |
| --- | --- | --- | --- | --- | --- | --- | --- | --- | --- | --- |
| **Outcome** | **Rurality** |  | **N** | **n** | **adj %** | **(95 % CI)** | **OR** | **(95 % CI)** | ***p^1^*** | ***p^2^*** |
| Overweight or obese (WHO) | MM2 | Regional Centres | 400 | 151 | 37.7 | (32.0 ,43.4) |  |  |  |  |
|  | MM3 | Large rural towns | 579 | 231 | 39.1 | (34.0 , 44.2) | 1.06 | (0.76 , 1.48) | 0.736 |  |
|  | MM4 | Medium rural towns | 354 | 126 | 33.8 | (28.0 , 39.7) | 0.84 | (0.58 , 1.22) | 0.357 |  |
|  | MM5-RA2 | Small rural towns - inner regional | 681 | 251 | 38.3 | (34.3 , 42.3) | 1.03 | (0.76 , 1.38) | 0.872 |  |
|  | MM5-RA3 | Small rural towns - outer regional | 316 | 84 | 29.8 | (23.4 , 36.1) | 0.70 | (0.47 , 1.03) | 0.073 | 0.126 |
| Vegetables (≥ 5 or 5.5 serves/day*) | MM2 | Regional Centres | 427 | 82 | 17.8 | (13.2 , 22.4) |  |  |  |  |
|  | MM3 | Large rural towns | 629 | 104 | 16.1 | (12.6 , 19.6) | 0.89 | (0.59 , 1.34) | 0.566 |  |
|  | MM4 | Medium rural towns | 371 | 55 | 14.5 | (10.3 ,18.7) | 0.78 | (0.49 , 1.26) | 0.308 |  |
|  | MM5-RA2 | Small rural towns - inner regional | 701 | 107 | 15.6 | (12.6 , 18.6) | 0.85 | (0.58 , 1.26) | 0.420 |  |
|  | MM5-RA3 | Small rural towns - outer regional | 323 | 56 | 17 | (11.6 , 22.5) | 0.95 | (0.58 , 1.55) | 0.832 | 0.871 |
| Fruit (≥ 2 serves/day) | MM2 | Regional Centres | 427 | 291 | 66.1 | (60.1 ,72.1) |  |  |  |  |
|  | MM3 | Large rural towns | 630 | 465 | 73.0 | (68.5 , 77.4) | 1.39 | (0.98 , 1.99) | 0.069 |  |
|  | MM4 | Medium rural towns | 370 | 245 | 65.4 | (59.2 , 71.6) | 0.97 | (0.66 , 1.43) | 0.872 |  |
|  | MM5-RA2 | Small rural towns - inner regional | 701 | 518 | 73.9 | (70.3 , 77.5) | **1.47** | **(1.05 , 2.04)** | **0.024** |  |
|  | MM5-RA3 | Small rural towns - outer regional | 323 | 271 | 84.9 | (80.2 , 89.6) | **2.95** | **(1.85 , 4.70)** | **<0.001** | **<0.001** |
| Water (≥ 5 glasses/day) | MM2 | Regional Centres | 427 | 226 | 51.9 | (45.9 , 57.9) |  |  |  |  |
|  | MM3 | Large rural towns | 630 | 360 | 56.6 | (51.7 , 61.5) | 1.21 | (0.88 , 1.66) | 0.239 |  |
|  | MM4 | Medium rural towns | 371 | 209 | 57.2 | (51.1 , 63.4) | 1.24 | (0.87 , 1.77) | 0.235 |  |
|  | MM5-RA2 | Small rural towns - inner regional | 700 | 391 | 55.5 | (51.4 , 59.6) | 1.16 | (0.86 , 1.55) | 0.327 |  |
|  | MM5-RA3 | Small rural towns - outer regional | 323 | 175 | 56.5 | (49.3 , 63.6) | 1.20 | (0.82 , 1.77) | 0.343 | 0.754 |
| Take away (≤once a fortnight) | MM2 | Regional Centres | 427 | 243 | 56.8 | (52.1 , 61.5) |  |  |  |  |
|  | MM3 | Large rural towns | 629 | 347 | 55.5 | (51.4 , 59.5) | 0.95 | (0.73 , 1.22) | 0.662 |  |
|  | MM4 | Medium rural towns | 371 | 219 | 59.9 | (54.7 , 65.0) | 1.14 | (0.85 , 1.52) | 0.395 |  |
|  | MM5-RA2 | Small rural towns - inner regional | 701 | 440 | 62.3 | (58.7 , 66.0) | 1.26 | (0.98 , 1.62) | 0.069 |  |
|  | MM5-RA3 | Small rural towns - outer regional | 323 | 231 | 71.2 | (65.6 , 76.7) | **1.89** | **(1.35 , 2.65)** | **<0.001** | **0.001** |
| Unhealthy snacks (< one/day) | MM2 | Regional Centres | 426 | 139 | 31.8 | (26.3 , 37.3) |  |  |  |  |
|  | MM3 | Large rural towns | 629 | 217 | 34.1 | (29.4 , 38.7) | 1.11 | (0.80 , 1.55) | 0.534 |  |
|  | MM4 | Medium rural towns | 371 | 120 | 34.4 | (28.3 , 40.5) | 1.13 | (0.77 , 1.65) | 0.528 |  |
|  | MM5-RA2 | Small rural towns - inner regional | 701 | 277 | 39.3 | (35.3 , 43.4) | **1.40** | **(1.03 , 1.90)** | **0.031** |  |
|  | MM5-RA3 | Small rural towns - outer regional | 323 | 162 | 50.8 | (43.7 , 58.0) | **2.25** | **(1.53 , 3.32)** | **<0.001** | **0.001** |
| Sweetened drinks (< one/day) | MM2 | Regional Centres | 427 | 235 | 57.4 | (50.5 , 64.3) |  |  |  |  |
|  | MM3 | Large rural towns | 629 | 328 | 53.4 | (47.7 , 59.0) | 0.84 | (0.58 , 1.24) | 0.384 |  |
|  | MM4 | Medium rural towns | 371 | 193 | 55.1 | (48.2 , 61.9) | 0.91 | (0.60 , 1.37) | 0.641 |  |
|  | MM5-RA2 | Small rural towns - inner regional | 701 | 425 | 59.3 | (54.9 , 63.7) | 1.09 | (0.77 , 1.53) | 0.642 |  |
|  | MM5-RA3 | Small rural towns - outer regional | 323 | 215 | 63.1 | (55.5 , 70.6) | 1.28 | (0.82 , 1.99) | 0.281 | 0.307 |

From logistic mixed models, school as random effect, adjusted for gender, grade and IRSAD tertile

MM: Modified Monash; RA: Remoteness Area (according to Accessibility/Remoteness Index Australia (ARIA+)); OR: Odds Ratio

Bold type indicates results that were significant at p<0.05.

^1^ Overall p-value

^2^ global p-value

**Supplementary Figure 1:** Adjusted percentages of children meeting food/drink consumption guidelines, by 5 level MMM_RA (N = 2,462)

*p<0.05 for odds ratio comparison (reference = Regional Centres)

- P<0.05

*

*

*

*

*
